# Magnetic Resonance Enterography of Phlebosclerotic Colitis: A Case Report

**DOI:** 10.2174/0115734056382571250509114555

**Published:** 2025-05-19

**Authors:** Yu-Xuan Kho, Chien-Ming Chen, Sung-Yu Chu

**Affiliations:** 1 Department of Medical Imaging and Intervention, Linkou Chang Gung Memorial Hospital, No. 5. Fuxing Street, Taoyuan 333, Taiwan; 2 College of Medicine, Chang Gung University, No. 5, De-Ming Road., Taoyuan 333, Taiwan

**Keywords:** Phlebosclerotic colitis, Ischemic colitis, Inflammatory bowel disease, Magnetic resonance enterography, Gardenia fruit, Case report, Biopsy

## Abstract

**Background:**

Phlebosclerotic colitis is a rare type of chronic ischemic colitis, with most documented cases occurring in Asians. Plain-film and computed tomography findings of pericolonic vascular calcifications are diagnostic. However, Magnetic Resonance Enterography (MRE) findings of phlebosclerotic colitis have not yet been reported, and its diagnosis might be overlooked without awareness of this disease.

**Case Report:**

A 70-year-old female patient without prior systemic disease presented with a 3-month history of nausea, vomiting, abdominal pain, and diarrhea. Personal history was unremarkable except for long-term use of herbal medicine. She was initially investigated at a regional hospital with a colonoscopy and biopsy. Due to the presence of stenosis at the transverse colon and biopsy results suggestive of Inflammatory Bowel Disease (IBD), she was referred to our hospital for further investigation and treatment. MRE was performed as part of the IBD workup, which showed a thickened ascending and transverse colonic wall that was fibrotic, non-edematous, and with triangular projections on the mesenteric aspect. Owing to findings that were inconsistent with IBD, subsequent abdominal plain-film radiography confirmed characteristic linear dendritic serpiginous radiopaque opacities alongside the ascending and transverse colon. Re-biopsy of the affected colon confirmed the diagnosis of phlebosclerotic colitis. The patient’s symptoms improved after conservative treatment.

**Conclusion:**

MRE of phlebosclerotic colitis appears as symmetrical non-edematous bowel wall thickening with triangular signal voids indicative of venous calcifications.

## 1 INTRODUCTION

Phlebosclerotic colitis is a rare form of chronic ischemic colitis, where most of the cases recorded are in individuals of Asian descent. Ischemic colitis occurs when blood flow to the colon is reduced, either due to arterial or venous issues. The exact pathogenesis of this disease is not yet known but is hypothesized to be due to the long-standing venous muscular injury, leading to progressive fibrosis and calcification, resulting in venous occlusion. Early phlebosclerotic colitis might not have calcifications [1]. Patients present with non-specific gastrointestinal symptoms, such as abdominal pain, diarrhea, nausea, and vomiting. Black and purplish colon mucosa on colonoscopy is one of the distinct entities of phlebosclerotic colitis [2].

## 2 CASE PRESENTATION

### 2.1 Chief Complaint

A 70-year-old Chinese female complained of nausea, vomiting, abdominal pain, and diarrhea for three months.

### 2.2 History of Present Illness

Symptoms persisted for three months without signs of improvement, accompanied by mild fatigue, general weakness, and poor appetite.

### 2.3 History of Past Illness

She denied any systemic disease, such as hypertension, diabetes mellitus, chronic renal disease, or previous operative history.

### 2.4 Personal and Family History

She mentioned a 15-year-long history of use of Chinese herbal medicine (Jiawei Siaoyao San Concentrated Preparation) and a habit of social drinking but denied a personal history of smoking and betel-nut chewing. She also denied a related family history.

### 2.5 Physical Examination

On physical examination, vital signs were as follows: temperature, 37.3°C; blood pressure, 135/85 mmHg; heart rate, 74 beats per minute; respiratory rate, 16 breaths per minute. There were no abnormal findings at the physical examination.

### 2.6 Laboratory Examination

Initial laboratory data showed no leukocytosis (8800/ μL) but showed a mildly increased percentage of segmented neutrophils (78.6%) and markedly elevated C-reactive protein (CRP) level up to 15.91 mg/L, suggestive of inflammation. The stool examination was 1+ for occult blood and unremarkable for other findings including for parasite and amebic infections. Amebiasis antibodies were equivocal. Further examination for amebiasis was negative. Her liver and renal functions were within normal range, while autoimmune markers, including ANA, anti-SMA, P-ANCA, C-ANCA, C3, and C4, showed no abnormal findings. Viral check-ups for HIV, CMV, EBV, and hepatitis were done. She turned out to be a hepatitis B carrier. There was no abnormality in urinalysis.

### 2.7 Imaging Examination

A colonoscopy performed at a regional hospital showed stenosis at the transverse colon, with biopsy results suggestive of Inflammatory Bowel Disease (IBD). She was referred to our hospital for further management.

## 3 FURTHER DIAGNOSTIC WORKUP

**Fig. (1):**
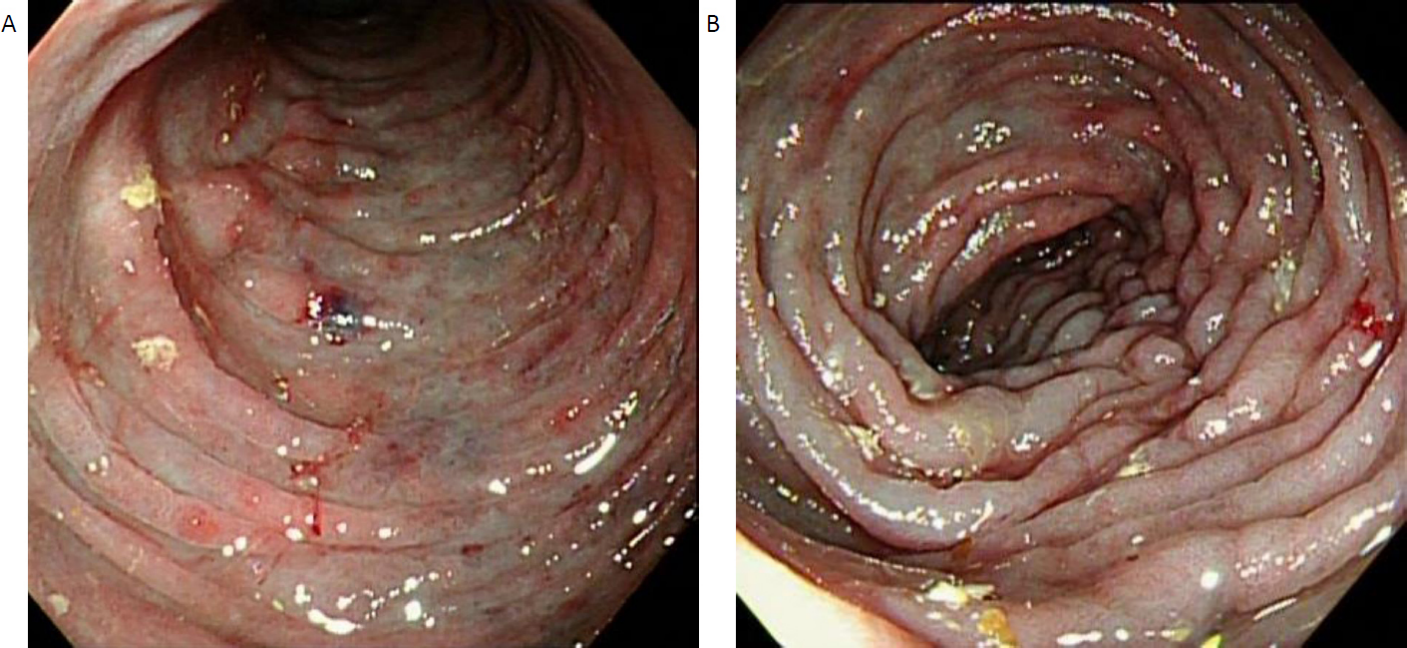
Colonoscopy shows (**A**) Black and purplish mucosal pigmentation at the transverse colon, (**B**) with easy bleeding ulcers and polypoid lesions along the examined colon passage.

Workup for IBD included colonoscopy and MR Enterography (MRE). Colonoscopy showed black mucosa with reddish spots with a hard base, easy bleeding ulcers and polypoid lesions, and stricture over the distal transverse colon (Fig. **1**). The colonoscope could not be advanced beyond the ascending colon. The affected segment was biopsied.

**Fig. (2):**
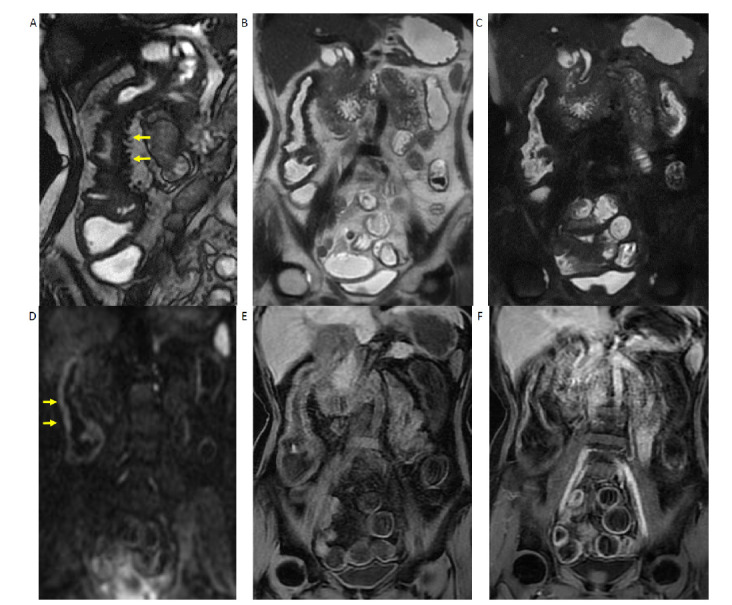
MR Enterography with 3% oral mannitol solution. (**A**) Fast imaging employing steady-state acquisition (FIESTA) sequence shows diffuse low signal intensity of the thickened ascending colon with multiple triangular signal voids along the mesenteric sides. (**B**) Fast spin-echo T2-weighted (FSE-T2W) and (**C**) fat-suppressed T2WI (FS-T2W) show no intramural edema and diffuse low signal intensity of ascending colon. Transverse colon is similarly affected; the terminal ileum is normal (not shown). (**D**) Diffusion-weighted images demonstrate moderate hyperintensity along the anti-mesenteric side of ascending colon. (**E**) Pre-contrast and (**F**) post-contrast T1-weighted images demonstrated no appreciable contrast enhancement at venous phase.

**Fig. (3):**
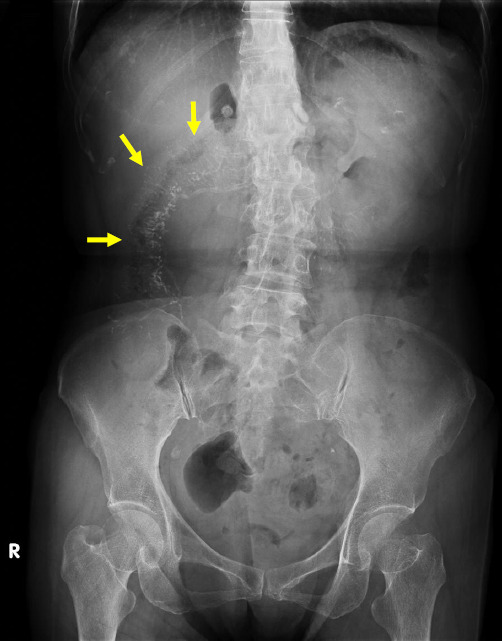
Abdominal plain film of the patient shows dendritic serpiginous radiopaque opacities alongside the ascending and transverse colon.

MRE with oral 3% mannitol solution showed symmetric and continuous wall thickening of the ascending and transverse colon with loss of haustrations (Fig. **2**). Luminal narrowing of the transverse colon was present, but there was no upstream bowel dilatation. The colon segments from the splenic flexure of the colon to the rectum were not affected. The terminal ileum and ileocecal valve were spared. Fast Imaging Employing a Steady-State Acquisition (FIESTA) sequence showed diffuse hypointensity of the thickened colon with triangular signal voids along the mesenteric side. Fat-suppressed T2-weighted images were likewise diffusely hypointense with no wall edema of the affected colon. However, there was moderate hyperintensity on diffusion-weighted images (*b* = 800) and moderate hypointensity on apparent diffusion coefficient maps predominately along the anti-mesenteric wall. There was no appreciable post-contrast enhancement of the thickened colon at arterial and venous phases. The MRE findings were not consistent with Crohn’s disease or ulcerative colitis. As the findings were indicative of ischemic changes of the ascending and transverse colon, and the triangular signal voids along the mesenteric side pointed to calcifications, an abdominal radiograph was requested to confirm the diagnosis of phlebosclerotic colitis. Abdominal plain-film revealed dendritic serpiginous calcifications along the mesenteric side of the ascending and transverse colon (Fig. **3**).

**Fig. (4):**
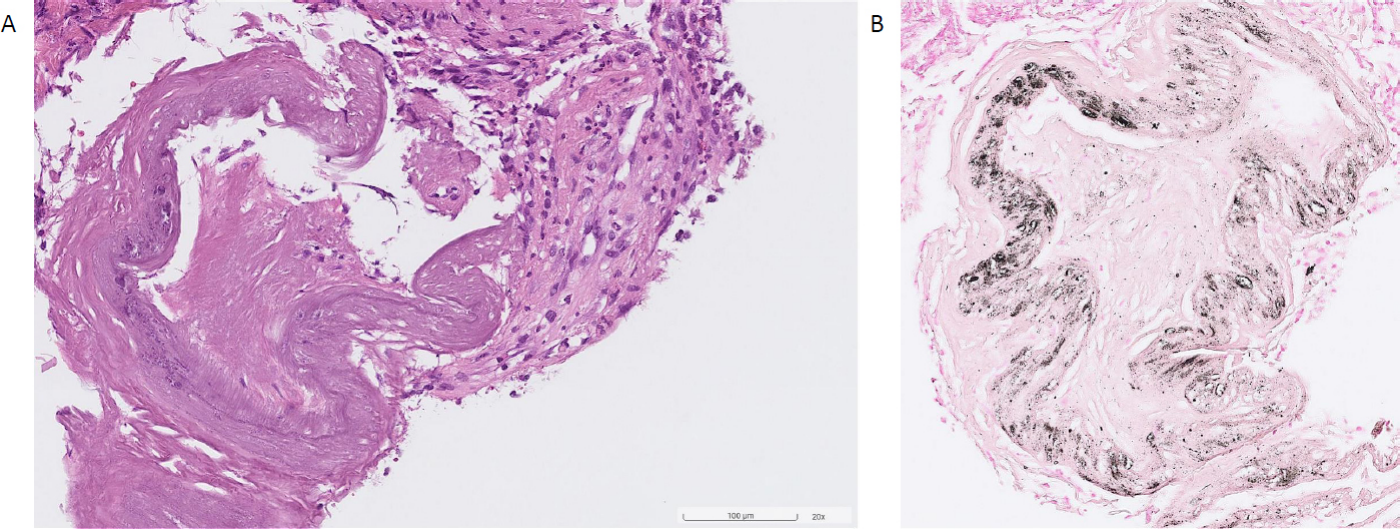
Pathology slides with (**A**) hematoxylin and eosin stain showed perivascular and lamina propria hyalinization with calcium deposits. (**B**) Von Kossa stain to detect abnormal calcium deposits.

Re-biopsy findings showed preserved crypt architecture, with eroded mucosa infiltrated predominately by neutrophils and eosinophils in the lamina propria (Fig. **4**). Perivascular and lamina propria hyalinization with calcium deposits in large vessels were present. Von Kossa stain confirmed the presence of perivascular calcium deposits. These findings were compatible with phlebosclerotic colitis.

**Fig. (5):**
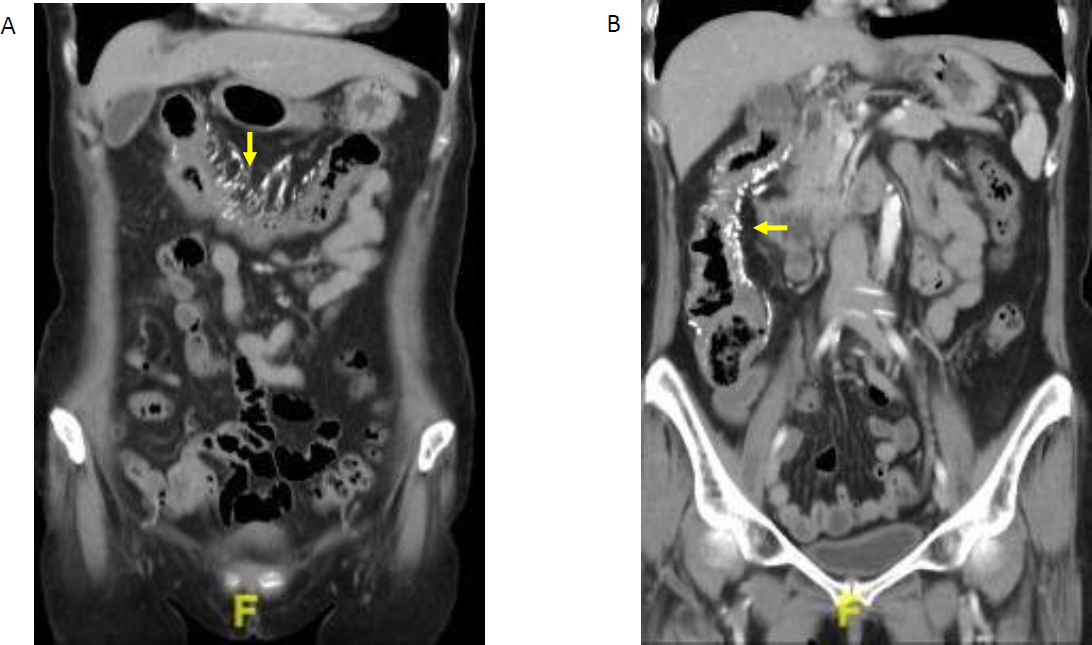
Computed tomography of the abdomen, coronal view, done at a regional hospital, shows characteristic vascular calcification of the pericolic arcades in the distribution of the (**A**) transverse and (**B**) ascending colons.

A subsequent review of abdominal computed tomography (CT) performed 2 months prior showed diffuse wall thickening of the ascending and transverse colon, loss of haustrations, and poor post-contrast enhancement (Fig. **5**). Characteristic vascular calcifications along the pericolic mesenteric veins were present.

## 4 FINAL DIAGNOSIS

Putting together the findings of her colonoscopy, MRE, abdominal plain-film radiography, CT, and histopathology results, the final diagnosis was phlebosclerotic colitis.

## 5 TREATMENT

The patient was admitted for diagnostic workups and received conservative treatment after confirming the diagnosis. Long-term use of Chinese herbal medicine (Jiawei Siaoyao San Concentrated Preparation) was thought to be the causative agent for phlebosclerotic colitis, so it was withheld after the diagnosis was made. Mesalazine granules and mosapride were prescribed for inflammation control and symptom relief.

## 6 OUTCOME AND FOLLOW-UP

The patient’s symptoms gradually improved with medical treatment. Follow-up laboratory data showed a decreasing trend of CRP level and gradually fell within the normal range (1.06 mg/L). The patient has regular outpatient follow-up, and her condition has remained stable until now, without progressive symptoms requiring admission or surgical treatment.

## 7 DISCUSSION

We present a patient with phlebosclerotic colitis, initially referred to our hospital as IBD. A general survey for diarrhea was done upon admission. Laboratory data were suggestive of inflammation status, the stool examination was negative for microorganisms or viral panel. Infectious disease was unlikely. The colonoscopy at the referral and our hospital were both incomplete as the ascending colon could not be explored due to stricture over the distal transverse colon.

While waiting for the formal histopathology report, MRE was carried out for suspected IBD. However, her MRE findings gave a different impression. Chronic ischemic colitis can mimic Crohn’s disease [3, 4]. In contradistinction to the classical findings of Crohn’s disease, such as asymmetrical wall thickening, intramural edema, and mural enhancement [5], the MRE of this patient showed symmetrical circumferential wall thickening of ascending and transverse colon without bowel edema [6]; T2-weighted images showed diffusely hypointense bowel wall. The terminal ileum and small bowel were normal. While Crohn’s disease affects any part of the gastrointestinal tract, it predominantly affects the small bowel, with the terminal ileum usually affected first [7]. Colonic ischemia more commonly occurs at the splenic flexure, which is the watershed area between the circulations of the superior mesenteric artery and inferior mesenteric artery [6] and is more associated with risk factors like atherosclerosis and vascular disease. In phlebosclerotic colitis, the right-sided colon is more likely involved [8], just like our patient with an affected ascending and transverse colon. Whereas in Crohn’s disease, active inflammation causes strong mural enhancement, in phlebosclerotic colitis, the absence of appreciable contrast enhancement suggests ischemia. Hypervascular appearance of mesentery named comb sign is often seen in active Crohn’s disease due to vasculitis [9].

In contradistinction, this patient had multiple telltale triangular lesions with signal voids at the mesenteric side of the affected colon on MRE that indicated calcifications. This finding on MRE was correlated with the dendritic serpiginous calcifications on her abdominal plain film and vascular calcifications of the pericolic arcade on CT.

Differential diagnoses for calcified, thickened colonic walls include lymphoma and colonic tuberculosis. Lymphoma involving the gastrointestinal tract typically presents as a mass-like lesion with eccentric wall thickening. Distal ileum is most commonly involved in B-cell lymphoma due to the presence of rich lymphoid tissue [10]. Diffuse B-cell proliferation can be noted histologically. Intestinal tuberculosis typically exhibits widespread involvement, most commonly in the ileocecal region, along with eccentric mural thickening and mesenteric lymphadenopathy. Tuberculosis of the colon predominantly affects the cecum and the ascending colon [11]. Strictures, inflammation, and polypoid lesions can be observed; however, these findings are non-specific [12]. Histopathology examination, M. tuberculosis culture, and AFB staining are done to check for intestinal tuberculosis [12].

The diagnostic CT was reviewed after completion of MRE because the CT was done in the previous regional hospital. A formal report from the previous hospital was not provided. Medical image sharing through the cloud is not well established in our country; thus the review of previous image studies was delayed.

Phlebosclerosis is the thickening and hardening of the venous wall by fibrous degeneration of the venous wall, predominantly the intima, with or without calcifications [13]. This process is thought to be the result of the adaptive change of the venous wall to the prolonged and increased venous blood pressure [14]. Our patient did not have a history of right-sided heart failure or portal hypertension, and this etiology cannot explain her phlebosclerosis.

However, early phlebosclerotic colitis does not necessarily present with pathognomonic dendritic calcifications along the colonic veins [1]. A non-Asian Spanish man with phlebosclerotic colitis experienced acute abdomen, and his CT study showed a few calcifications along the colonic wall [15]. The detailed pathogenesis, including the temporal changes, has yet to be clarified. Yen *et al.* [16] studied the association between the extent of venous calcifications and the severity of clinical symptoms in phlebosclerotic colitis. Although the study group was small (*n* = 12), the results strongly suggested that the extent of mesenteric venous calcification is positively associated with the number of episodes of active disease. Calcification of the mesenteric venous system causes venous congestion of the colon, which leads to chronic ischemia of the colon [17].

Previous publications concluded that phlebosclerotic colitis is highly relevant to long-term herbal medicine use or some ingested materials that metabolize primarily in the colon [17-19]. More recent studies analyzed patients with long-term herbal medicine use. Xu *et al.* [20] reviewed the medication use of 10 patients with phlebosclerotic colitis; they all had a history of long-term use of Chinese medicine liquor or traditional Chinese medicine.

Gardenia fruit was one of the ingredients of the Chinese medicine liquor. Gardenia fruit has been found to have a remarkable antithrombotic effect. Studies have also shown that it decreases systolic blood pressure, along with a reduction in cardiac output and stroke volume in rats [21].

A nationwide survey of phlebosclerotic colitis in Japan collected data from 222 patients, of which 147 had a history of herbal medicine use [22]. A total of 119 out of 147 used herbal medicine containing *sanshishi* (gardenia fruit, *Gardenia jasminoides* Ellis). Gardenia fruit is thought to be one of the causative agents of phlebosclerotic colitis. Geniposide is the main active material of Gardenia fruit. The metabolite of geniposide, genipin, is more toxic than geniposide. Orally ingested geniposide is hydrolyzed and metabolized by β-glucosidase after entering the cecum and ascending colon [23, 24]. This explains the predominance of phlebosclerotic colitis in the right colon.

The metabolite permeates the enterocyte membrane and reacts with amino acids/proteins in mesenteric vein plasma. Collagen accumulation under the mucosa slowly progresses to hyperplasic myointima in the veins, fibrosis, and sclerosis. These changes result in venous occlusion [25]. Genipin turns dark blue when it comes in contact with amino acids. Venous occlusion and the chemical reaction between genipin and amino acids are thought to contribute to the purple colonic mucosa [26]. The medication that our patient had been using for 15 years had gardenia fruit as one of the main ingredients. We can boldly say that the phlebosclerosis of our patient was due to long-term medication use. Although phlebosclerotic colitis is irreversible [1], her symptoms improved upon the withdrawal of the herbal medication.

Gardenia fruit is a type of shrub found in tropical and subtropical regions. It is widely used as a food colorant and in traditional Chinese medicine in Eastern countries [27], which may explain the higher prevalence of phlebosclerotic colitis among Asians. Therefore, most studies on phlebosclerotic colitis to date have focused on Asian populations.

A long-term follow-up study on 29 patients with phlebosclerotic colitis of varying severity was conducted, and only 6 patients used herbal medicine. According to Ko *et al.*, concurrent renal and hepatic failure could be among the risk factors for phlebosclerotic colitis [28]. As non-Asian cases have been reported, genetic predisposition should also be considered as one of the causative factors of phlebosclerotic colitis.

Dark purple colonic mucosa at colonoscopy is a distinctive finding of phlebosclerotic colitis [6]. Besides the characteristic mucosal pigmentation change, extensive erosion, ulceration, multi-focal nodular surface, and luminal narrowing manifest and can be seen as the disease progresses [29, 30]. Typically, endoscopic findings in Crohn’s disease include discontinuous distribution of longitudinal ulcers, cobblestone appearance, and/or small aphthous ulcerations arranged in a longitudinal fashion, fistulous orifices, and strictures [31]. Endoscopic features of lymphoma typically include a distinctive large ulcerated mass, though polypoid appearances and, on rare occasions, an annular napkin-ring lesion may also be observed [32]. The key endoscopic features of colonic tuberculosis include ulceration, nodularity, and stricture [11].

Our case report has some limitations. As phlebosclerotic colitis is rare and serpiginous calcifications along colonic veins are pathognomonic, this disease is easily diagnosed with plain films or CT. The findings from our case may not be seen in other phlebosclerotic colitis cases. Additionally, there is a lack of long-term follow-up in terms of imaging, as MR studies are costly, and due to the insidious nature of this disease, follow-up imaging studies are expected to show changes years later.

## CONCLUSION

Phlebosclerotic colitis is a chronic and insidious type of ischemic colitis that occurs mostly in Asians. Diagnostic imaging studies typically reveal calcifications in pericolic vascular arcades, although early-stage phlebosclerotic colitis might not exhibit such calcifications. Distinctive purple mucosa at endoscopy is a characteristic feature of the disease. MRE findings include a symmetrically thickened colon wall that appears diffusely hypointense on T2-weighted images, triangular signal voids along the mesenteric side of the colon, and no significant contrast enhancement. Radiologists should be aware of this rare entity and consider it when providing differential diagnosis, taking care not to confuse it with conditions such as Crohn’s disease.

## Data Availability

All data generated or analyzed during this study are included in this published article.

## LIST OF ABBREVIATIONS

IBD: Inflammatory Bowel Disease
MRE: MR Enterography
CRP: C-reactive protein
FIESTA: Fast Imaging Employing a Steady-State Acquisition

## STANDARDS OF REPORTING

CARE guidelines were followed.
